# Geriatric nutritional risk index predicts prognosis after hepatectomy in elderly patients with hepatitis B virus-related hepatocellular carcinoma

**DOI:** 10.1038/s41598-018-30906-8

**Published:** 2018-08-22

**Authors:** Lei Li, Haiqing Wang, Jian Yang, Li Jiang, Jiayin Yang, Hong Wu, Tianfu Wen, Lvnan Yan

**Affiliations:** 1 Department of Liver Surgery & Liver transplantation center, West China Hospital of Sichuan University, Chengdu, China; 2 Department of Hepato-pancreato-biliary Surgery, Sichuan cancer hospital, Chengdu, China

## Abstract

Geriatric nutritional risk index (GNRI) is a novel and useful screening tool for evaluating nutritional status in elderly in-patients. We aimed to investigate whether the preoperative GNRI could be a predictive factor for outcomes in patients over 65 years of age with a diagnosis of hepatocellular carcinoma (HCC). We retrospectively enrolled 261 consecutive HCC patients after hepatectomy and classified them into four risk groups based on the GNRI values: high risk (GNRI, <82), moderate risk (GNRI, 82–92), low risk (GNRI, 92–98), and normal (GNRI, >98). We found that the lower GNRI value was significantly associated with severe postoperative complications (*P* < 0.001) and liver failure (*P* < 0.001). By multivariate logistic regression analysis, high risk- and moderate risk GNRI groups were identified as independent risk factors for postoperative serve complications and liver failure. Multivariate Cox regression analysis revealed preoperative GNRI (*P* < 0.001) adversely affected overall survival. In conclusion, preoperative GNRI could predict severe postoperative complications included liver failure, and the lower GNRI value was associated with worse overall survival after hepatectomy in elderly HCC patients.

## Introduction

Liver cancer is the fourth leading cause of cancer-related deaths according to the Global Burden of Disease Study in 2015^1^. The most common type of primary liver cancer is hepatocellular carcinoma, followed by cholangiocarcinoma^2^. Partial hepatectomy is the preferred curative treatment for patients with HCC. However, malnutrition may contribute to an increase in surgical risk and prolonged hospital stays and was shown to significantly increase postoperative morbidity and mortality in the elderly^3,4^. In older in-patients, malnutrition is associated with poor clinical outcomes, including impairment in quality of life, performance status, immune function and liver function, as well as decreased survival. Several studies^5–7^ reported a relationship between preoperative nutritional status and poor prognosis in HCC patients. However, the correlation between preoperative nutritional status and prognosis in elderly patients with HCC remains unclear and deserves investigation.

Geriatric nutritional risk index, a new prognostic nutritional index, has been proposed for evaluation of at risk, in-hospital, elderly patients with malignant tumors such as non-small cell lung cancer^8–11^, renal cell carcinoma^12,13^, and esophageal squamous cell carcinoma^14,15^. The relationship between GNRI and prognosis in patients with HCC after hepatectomy has not yet been reported.

Thus, we conducted a cohort study to investigate the relationship between GNRI and short-, long-term outcomes for elderly HCC patients after hepatectomy.

## Materials and Methods

### Study population

We included 261 consecutive HCC patients who underwent hepatectomy in West China hospital, Sichuan university between February 2009 and December 2012. Preoperative diagnosis was confirmed based on the current EASL^16^ or AASLD^17^ HCC management guidelines. In the present study, the inclusion criteria followed: (1) Pathological diagnosis confirmed hepatocellular carcinoma, (2) receiving radical resection by open operation as the initial treatment, (3) chronic HBV infection history, (4) elderly patients >65 years old. Exclusion criteria included the following: (1) patients with obstructive jaundice, (2) combined with portal vein tumor thrombus, (3) combined with extrahepatic metastasis, (4) inpatients received albumin infusion for preoperative severe hypoproteinemia, (5) liver function of Child-Pugh grade B, (6) loss to postoperative follow-up within 90 days, (7) poor data integrity. We collected the medical records containing patients’ demographics, preoperative laboratory values, imaging examination data and postoperative clinical outcomes from the clinical liver cancer database of the department of Liver Surgery & Liver Transplantation Center, West China Hospital, Sichuan university.

This study was approved by the clinical research ethics committee of the West China Hospital, Sichuan University and performed in accordance with the principles of the Declaration of Helsinki. Written informed consent was obtained from all patients according to the policies of the committee.

### Perioperative management

Careful historical analysis, physical examination and routine preoperative laboratory measurements were performed for all patients. Routine preoperative imaging examination to evaluate the tumor and preoperative cardiopulmonary function evaluation were carried out before surgery as we previously described^18^. Patients were operated under general anesthesia and intraoperative ultrasonography was used routinely. Hepatic vascular inflow occlusion (hemihepatic or total hepatic blocking) or the Pringle maneuver was used according to the surgeon’s preference in most patients as those previously described^19,20^. Hepatectomy was performed using the clamp crushing, hooking with ligation or ultrasonic dissector with coagulator. Based on preoperative and intraoperative condition, patients were transferred to the intensive care unit for treatment when necessary.

### Parameters definition

The Clavien-Dindo complication classification^21^ system was used for postoperative complication grading. Postoperative liver failure was defined according to the criterion of International Study Group of Liver Surgery^22^. Perioperative mortality was defined as any death occurring from the time of surgery up to 90 days after discharge. The GNRI is, an adaptation of the Nutritional Risk Index (NRI) designed by Buzby *et al*.^23^, simple nutritional screening tools to estimate nutrition related risk in surgical patients. The formula is as follows: GNRI = 1.487 × serum albumin concentrations (g/L) + 41.7 × preoperative body weight/ideal body weight (kg). Serum albumin levels were measured in a fasting state, blood samples were collected 3–5 days before surgery. Ideal body weight = 22 × squares of height (m), which calculated using Lorentz equations. As in previous reports^24,25^, the patients were classified as at high risk (GNRI < 82), moderate risk (82–92), low risk (92–98), and normal level (GNRI > 98).

### Follow-up

All include patients were received regularly in the out-patient clinic and monitored prospectively by a standard protocol; the follow-up program was same as the previously we used (Follow-up time point: 30 days after the operation, every 90 days thereafter during the first 3 years and then every 180 days in subsequent years)^26^. HCC recurrence was diagnosed by clinical, laboratory examination and radiological data at each follow up. They were followed up until December 2017 or their death, and their medical records were retrospectively reviewed. Overall survival (OS) time was described as the interval between the operation and death or the last follow up. Recurrence-free survival (RFS) time was described as the interval between the operation and the first incidence of HCC recurrence.

### Statistical analysis

Scientific secretaries were trained to take advantage of the collection and analysis responsibilities. Continuous variables were reported as mean (standard deviation [SD]) or median (interquartile range [IQR]), and the comparation using the Student t test for continuous variables with parametric distribution. Mann–Whitney U test or Kruskal–Wallis H test for those with nonparametric distribution. Categorical variables were recorded as numbers and percentages, and compared using Pearson *x*^2^ analysis or Fisher exact test. To identify risk factors for postoperative liver failure or severe complications, only significant factors in the univariate analysis were entered into the forward stepwise Logistic regression analysis. Independent risk factors for overall survival were identified using the stepwise forward Cox regression model. Overall survival- and recurrence-free survival curves were plotted using the Kaplan-Meier method and analyzed using the Log-rank test. All statistical analyses were performed using IBM SPSS Statistics software 21.0 and GraphPad Prism 7.00. *P* < 0.05 was considered as statistically significant.

## Results

### Patient characteristics

This study comprised a total of 261 patients including 215 (82.4%) males and 46 (17.6%) females. The age of the patients was 69.0 ± 3.4 years, and average total tumor size was 4.9 ± 2.2 cm. In total, 102 (39.1%) patients with HCC met the Milan criteria, whereas the remaining 159 (60.9%) patients exceeded. The preoperative liver function were classified as Child-Pugh A in all included patients. A total of 41 (15.7%) patients developed postoperative liver failure (PLF), 32 (12.2%) patients were classified as PLF grade A and B. Severe postoperative complications occurred in 19 (7.3%) cases included liver failure grade C (9 cases), cardiovascular and pulmonary complications (5 cases), bile leakage (3 cases), intra-abdominal infections (2 cases). no patient dead in all cases. The length of hospital stay after operation was 9.8 ± 3.2 days. Preoperative albumin level was significantly lower in the high-risk group than in the other three groups (*P* < 0.001). Incidence of severe postoperative complications (5/9, 55.5%) and liver failure (6/9, 66.6%) were significantly higher in the high-risk group (*P* < 0.001 for both). The details are presented in Table 1.

**Table 1:** The characteristics and clinical parameters in the four groups based on the GNRI values.

Variables	Total	High risk	Moderate risk	Low risk	Normal Level	*P value*
		GNRI < 82	GNRI 82–92	GNRI 92–98	GNRI > 98	
Patients (n,%)	261, 100%	9, 3.4%	17, 6.5%	38, 14.6%	197, 75.5%	
Age, median (IQR)	68 (67–70)	69 (68–72)	70 (66–72.5)	68 (66–70.1)	68 (67–70)	0.330
Preoperative TBIL (umol/L), median (IQR)	14.1 (10.8–18.7)	16.2 (12.5–27.1)	17.0 (10.9–19.3)	15.4 (10.9–19.6)	13.3 (10.8–18.4)	0.415
Preoperative ALB (g/L), median (IQR)	41.4 (38.1–43.8)	28.1 (24.5–30.1)^#^	34.7 (31.0–36.3)	37.5 (35.9–38.8)	42.7 (40.2–44.6)	<0.001
Preoperative platelet (10^9^/L), median (IQR)	132 (91–182)	133 (86.5–234.5)	145 (123–173)	105 (75.0–160.3)	138 (91–185)	0.280
Total diameter of tumor (cm), mean (SD)	4.9 ± 2.2	4.7 ± 2.2*	5.8 ± 2.2	5.8 ± 2.6	5.3 ± 1.8	0.009
Tumor number (single/multiple), median (IQR)	186/75	3/6	6/11	8/30	58/139	0.661
Preoperative AFP > 400 ng/mL (Y/N)	184/77	4/5	6/11	14/24	53/144	0.413
Positive HBV-DNA load (Y/N)	70/191	4/5	5/12	12/26	49/148	0.512
Preoperative GNRI, median (IQR)	103.4 (98.1–109.7)	78.4 (76.9–80.0)^#^	88.7 (86.3–90.5)	94.9 (93.5–96.9)	106.2 (103.4–112.4)	<0.001
Ishak score, median (IQR)	6 (5–6)	6 (5–6)	6 (5–6)	6 (5–6)	6 (4–6)	0.520
Presence of MVI (Y/N)	29/232	0/9	2/15	6/32	21/176	0.569
Differentiation						0.274
Well (n,%)	28, 10.7%	1,0.3%	1,0.3%	4,1.5%	22,8.4%	
Moderate (n,%)	218, 83.5%	7,2.7%	13,4.9%	34,13.0%	164,62.8%	
Poor (n,%)	15, 5.7%	1,0.3%	3,1.1%	0,0%	11,4.2%	
Transfusion (Y/N)	42/219	3/6	2/15	8/30	29/168	0.364
Severe complication (Y/N)	19/261	5/9^#^*	4/17	0/38	10/197	<0.001
Liver failure (Y/N)	41/261	6/9^#^*	9/17	6/38	20/197	<0.001
Postoperative hospital stay (day), median (IQR)	9 (8–11)	10 (8–16)	12 (8–16)	9 (8–10)	9 (7–11)	0.090

GNRI = Geriatric Nutritional Risk Index, AFP = alpha-fetoprotein, TBIL = total bilirubin, ALB = serum albumin, PLT = platelet, MVI = micro-vascular invasion, IQR interquartile range, SD standard deviation.

^#^*P* < 0.05, when High risk group vs. Normal level-, Moderate risk- or Low risk-group.

^*^P < 0.05, when High risk group vs. Moderate risk- or Low risk-group.

### Risk factors for severe postoperative complications and liver failure

**Figure 1:**
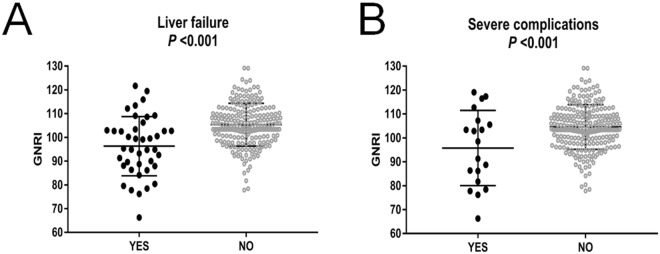
Incidence of liver failure **(A)** and severe complications **(B)** after hepatectomy according to GNRI. Mean GNRI was 96.29 ± 1.94 in patients who occurred postoperative liver failure (n = 41), mean GNRI was 105.3 ± 0.61 in whose without (n = 220), the significant differences between the two groups. **(B)** Mean GNRI was 95.76 ± 3.61 in patients (n = 19) who occurred postoperative complication, mean GNRI was 104.6 ± 0.6 in whose without (n = 242), the significant differences between the two groups.

The GNRI was significantly higher in patients without than those with postoperative complications (Fig. 1). Multivariate logistic regression analysis revealed that preoperative GNRI value (hazard ratio [HR] 0.910, 95% confidence interval [CI] 0.876–0.945, *P* < 0.001), moderate risk (HR 9.956, 95%CI 3.454–28.699, *P* < 0.001) and high-risk groups (HR 17.700, 95%CI 4.106–76.291, *P* < 0.001) were risk factors for postoperative liver failure (Table 2). Moderate-risk (HR 8.726, 95%CI 2.130–35.752, *P* = 0.003) and high-risk groups (HR 26.336, 95%CI 5.576–124.383, *P* < 0.001) and transfusion (HR 0.161, 95%CI 0.046–0.560, *P* = 0.004) were identified as independent risk factors for severe postoperative complications (Table 3).

**Table 2:** Univariate and multivariate analyses of prognostic factors for postoperative liver failure in elderly patients with HCC.

Variables	Univariate analysis			Multivariate analysis		
	HR	95%CI	*P* value	HR	95%CI	*P* value
Age (year)	1.001	0.892–1.122	0.992			
Child-Pugh score	1.858	0.904–3.819	0.092			
Preoperative TBIL (umol/L)	0.999	0.990–1.008	0.827			
Preoperative ALT (IU/L)	0.997	0.986–1.007	0.520			
Preoperative AST (IU/L)	1.007	0.997–1.017	0.193			
Preoperative ALB (g/L)	0.979	0.880–1.089	0.695			
Preoperative platelet (10^9^/L)	1.004	0.998–1.010	0.189			
Preoperative AFP > 400 ng/mL (Y/N)	1.317	0.572–3.030	0.517			
Positive HBV-DNA load (Y/N)	1.380	0.541–3.517	0.500			
Preoperative GNRI	0.918	0.865–0.975	0.005	0.910	0.876–0.945	<0.001
**Preoperative GNRI grade**						
Normal Level (GNRI > 98)	1(Reference)			1(Reference)		
Low risk (GNRI 92–98)	1.547	0.476–5.035	0.468			
Moderate risk (GNRI 82–92)	9.164	2.141–39.233	0.003	9.956	3.454–28.699	<0.001
High risk (GNRI < 82)	11.07	1.289–95.083	0.028	17.700	4.106–76.291	<0.001
Total diameter of tumor (cm)	0.894	0.737–1.085	0.256			
Tumor number (single/multiple)	0.486	0.186–1.268	0.140			
Transfusion (Y/N)	0.538	0.197–1.467	0.226			

GNRI = Geriatric Nutritional Risk Index, AFP = alpha-fetoprotein,TBIL = total bilirubin. AST = aspartate aminotransferase, ALT = alanine aminotransferase, ALB = serum albumin. PLT = platelet.

**Table 3:** Univariate and multivariate analyses of prognostic factors for postoperative severe complications in elderly patients with HCC.

Variables	Univariate analysis			Multivariate analysis		
	HR	95%CI	*P* value	HR	95%CI	*P* value
Age (year)	1.119	0.822–1.523	0.474			
Child-Pugh score	2.532	0.970–6.611	0.058			
Preoperative TBIL (umol/L)	1.000	0.981–1.018	0.967			
Preoperative ALT (IU/L)	1.004	0.990–1.019	0.573			
Preoperative AST (IU/L)	0.998	0.985–1.012	0.822			
Preoperative ALB (g/L)	0.957	0.817–1.122	0.591			
Preoperative platelet (10^9^/L)	0.996	0.986–1.006	0.431			
Preoperative AFP > 400 ng/mL (Y/N)	2.993	0.904–9.911	0.073			
Positive HBV-DNA load (Y/N)	4.040	0.839–1.464	0.082			
Preoperative GNRI	0.919	0.849–0.994	0.036			
Preoperative GNRI grade						
Normal Level (GNRI > 98)	1(Reference)			1(Reference)		
Low risk (GNRI 92–98)	0.000	0.000	0.998			
Moderate risk (GNRI 82–92)	5.754	1.586–20.874	0.008	8.726	2.130–35.752	0.003
High risk (GNRI < 82)	23.375	5.425–100.712	<0.001	26.336	5.576–124.383	<0.001
Ishak score	1.000	0.516–1.938	0.999			
Total diameter of tumor (cm)	1.105	0682–1.792	0.684			
Tumor number (single/multiple)	0.192	0.050–0.745	0.017			
Transfusion (Y/N)	0.180	0.045–0.718	0.015	0.161	0.046–0.560	0.004

GNRI = Geriatric Nutritional Risk Index, AFP = alpha-fetoprotein,TBIL = total bilirubin.

AST = aspartate aminotransferase, ALT = alanine aminotransferase, ALB = serum albumin.

PLT = platelet.

### Long-term outcomes: overall survival and HCC recurrence

**Figure 2:**
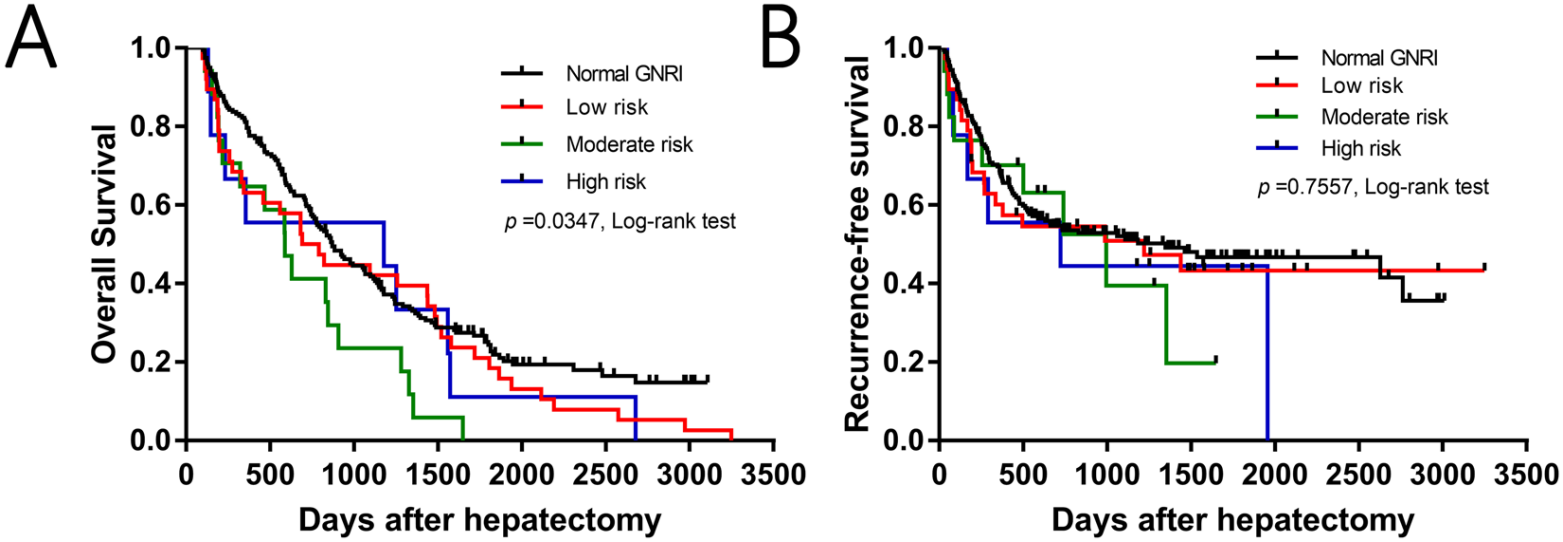
Kaplan-Meier curve analysis of Overall survival **(A)** and Recurrence-free survival **(B)** in four groups based on GNRI values.

Based on the classification of patients according to GNRI values, 1-, 3-, and 5-year overall survival rates were significantly different among the high-risk (79.6%, 42.4%, and 22.7%, respectively), moderate-risk (63.2%, 42.1%, and 18.4%, respectively), low-risk (64.7%, 23.5%, and 0%, respectively), and normal (55.6%, 55.6%, and 11.1%, respectively) GNRI groups (*P* = 0.0347, Fig. 2A). However, the 1-, 3-, and 5-year recurrence-free survival rates were not significantly different among the high-risk (67.7%, 52.1%, and 46.7%, respectively), moderate-risk (60.1%, 50.9%, and 43.3%, respectively), low-risk (70.1%, 39.4%, and 19.7%, respectively), and normal (55.6%, 44.4%, and 44.4%, respectively) GNRI groups (*P* = 0.7557, Fig. 2B). To identify independent risk factors for overall survival, univariate and multivariate analysis was performed, which revealed that preoperative platelet counts (HR 1.003, 95%CI 1.001–1.005, *P* = 0.001), microvascular invasion (HR 0.634, 95%CI 0.414–0.971, *P* = 0.036), and preoperative GNRI value (HR 0.977, 95%CI 0.964–0.990, *P* < 0.001) were independent prognostic factors for overall survival (Table 4).

**Table 4:** Univariate and multivariate analyses of prognostic factors for overall survival in elderly patients with HCC.

Variables	Univariate analysis			Multivariate analysis		
	HR	95%CI	*P* value	HR	95%CI	*P* value
Age (<70/ ≥ 70)	1.145	0.831–1.578	0.408			
Milan Criteria (within/exceed)	0.700	0.500–0.981	0.039			
Total diameter of tumor (cm)	1.037	0.922–1.167	0.541			
Child-Pugh score	1.095	0.874–1.372	0.430			
Preoperative platelet (10^9^/L)	1.003	1.001–1.005	0.003	1.003	1.001–1.005	0.001
Preoperative AFP > 400 ng/mL (Y/N)	0.762	0.540–1.077	0.124			
Positive HBV-DNA load (Y/N)	0.958	0.690–1.331	0.799			
Preoperative GNRI	0.981	0.963–0.999	0.039	0.977	0.964–0.990	< 0.001
Preoperative GNRI grade						
Normal Level (GNRI > 98)	1(Reference)					
Low risk (GNRI 92–98)	1.265	0.881–1.816	0.202			
Moderate risk (GNRI 82–92)	2.003	1.208–3.322	0.007			
High risk (GNRI < 82)	1.313	0.669–2.577	0.428			
Ishak score	1.074	0.907–1.273	0.408			
Tumor number (single/multiple)	1.457	1.030–2.062	0.034			
Presence of MVI (Y/N)	0.623	0.398–0.976	0.039	0.634	0.414–0.971	0.036
Differentiation	1.501	0.731–3.081	0.269			
Well	1(Reference)					
Moderate	0.703	0.461–1.071	0.101			
Poor	0.679	0.341–1.353	0.271			
Transfusion (Y/N)	0.903	0.601–1.356	0.623			

GNRI = Geriatric Nutritional Risk Index, AFP = alpha-fetoprotein, TBIL = total bilirubin.

AST = aspartate aminotransferase, ALT = alanine aminotransferase, ALB = serum albumin.

PLT = platelet, MVI = micro-vascular invasion.

## Discussion

The postoperative complications of hepatectomy include intractable ascites, bile leakage, intra-abdominal hemorrhage, and liver failure. The incidence of postoperative liver failure, reported to be as high as 5.7%–11%, was reported as a predominant cause of hepatectomy-related mortality with incidence rates as high as 11%^22^. The complications are widely known to significantly increase the risk of postoperative morbidity and mortality and to have a negative impact on long-term survival.

Malnutrition is associated with worse outcome of partial hepatectomy and appropriate nutritional intervention can improve the outcomes. Previous studies suggested that nutritional supplementation could reduce the postoperative complications and shorten the duration of hospitalization of patients who undergo liver resection for cancer^27,28^. Hsieh CE *et al*. reported that postoperative nutritional support could promote the recovery of liver function and shorten length of stay in adult liver donors^29^.

This is the first study to retrospectively investigate the correlation between preoperative nutritional status using GNRI and prognosis in elderly patients with HCC, which revealed that lower preoperative GNRI value in elderly HCC patients were associated with worse postoperative clinical outcomes, such as liver failure, severe complications, and overall survival rate, but not HCC recurrence-free survival. Although most of the patients appeared to be in good health before the operation, the true malnutrition status and acceptable organ functions were often ignored. The results of the current study demonstrated that preoperative GNRI was an independent predictive factor for prognosis after hepatectomy in elderly patients with HCC.

Changes in physiologic and psychosocial factors resulting in malnutrition were found to increase risk in adults over the age of 65 in a study conducted in the United States^4^. Malnutrition is associated with an increase in the risk of operation and prolonged hospital stays and markedly contributes to morbidity and mortality in the elderly. Our multivariate logistic regression analysis revealed that GNRI was an independent risk factor for both postoperative liver failure and severe postoperative complications. We also found that platelet count, microvascular invasion, and GNRI were independent risk factors for overall survival analyzed by Cox proportional hazard model. However, for the recurrence-free survival, GNRI was not an independent risk factor in the same analysis. We previously reported that^30^ postoperative liver failure was significantly associated with low platelet counts. Consistent with previous studies^31,32^, postoperative liver failure and severe complications affected HCC recurrence and reduced overall survival. The presence of microvascular invasion was shown to be associated with a high incidence of recurrence and worse long-term survival in many studies^33–35^. Bo *et al*.^14^ indicated that the GNRI could predict survival in elderly esophageal cancer patients. Gu *et al*.^13^ found that GNRI could be utilized to identify patients with metastatic renal cell carcinoma at risk for poor survival outcomes. Shoji *et al*.^9^ reported that elderly patients with non-small cell lung cancer and abnormal preoperative GNRI experienced significantly shorter overall survival. These earlier studies lend further support to our findings in the current study.

Chronic cardiopulmonary disease, hypertension, and glucose and lipid metabolism disorders, which occur at a higher incidence in the elderly population, could affect the BMI values or albumin level in elderly patients. The BMI consists of body weight and height, which is related to malnutrition^36^. In the study, ascites was found in 62 patients via the preoperative ultrasound examination and no patients complained of abdominal distention. The shifting dullness was negative. The depth of ascetic fluid under ultrasonic examination is not more than 3 cm. In addition, mean (SD) and median (IQR) preoperative albumin levels were 40.79 ± 5.09 and 41.40 (38.05–43.80), respectively. No patients received albumin infusion before operation. Previous studies found that the influence of BMI on the postoperative course and survival have shown controversial results^37,38^ and albumin could assess the nutritional status and predict long-term mortality of elderly patients^39^. The authors considered that the GNRI, with the additional information for ideal weight, might predict nutrition-related mortality better than serum albumin or BMI.

The current study has several limitations that should be acknowledged. First, potential information and selection biases cannot be denied in this retrospective, single-center study. Second, the definition of the elderly population is not consistent among studies, and these analyses should also be performed in patients above 70 or even 80 years of age. Third, only GNRI was used as the nutritional screening tool, and GNRI was not compared with other commonly utilized tools to assess nutritional status, which should be addressed in future studies.

In conclusion, this retrospective study revealed that preoperative GNRI could predict severe postoperative complications included liver failure and the lower GNRI score was associated with worse overall survival after hepatectomy in elderly patients with HCC.

### Ethical Review

This protocol was approved by the West China Hospital Ethical Committee and written informed consents were obtained from all the patients before their operation.

## Data Availability

The data-sets used and/or analyzed during the current study are available from the corresponding author on reasonable request.
